# Experience and measurement of fatigue in adults with Crohn’s disease: results from qualitative interviews and a longitudinal 2-week daily diary pilot study

**DOI:** 10.1186/s41687-023-00612-9

**Published:** 2023-07-20

**Authors:** Miguel Regueiro, Laure Delbecque, Theresa Hunter, Larissa Stassek, Gale Harding, James Lewis

**Affiliations:** 1 Cleveland Clinic, Cleveland, OH USA; 2 Eli Lilly and Company, 893 Delaware St, Indianapolis, IN 46225 USA; 3 Evidera, Bethesda, MD USA; 4 Perelman School of Medicine, University of Pennsylvania, Philadelphia, PA USA

**Keywords:** Construct validity, Functional Assessment of Chronic Illness Therapy–Fatigue, Patient-reported outcome, Psychometric properties

## Abstract

**Background:**

Fatigue has a detrimental impact on health-related quality of life and functioning in patients with Crohn’s disease (CD). We aimed to confirm the relevance and importance of fatigue, establish the content validity of the Functional Assessment of Chronic Illness Therapy–Fatigue (FACIT-F), qualitatively explore meaningful change in fatigue experience, and assess the measurement properties of the FACIT-F in patients with moderate-to-severe CD.

**Methods:**

This was a mixed-methods observational study consisting of a cross-sectional qualitative interview (Part A) and a longitudinal 2-week daily diary pilot study (Part B) in participants aged ≥ 18 years with a clinical diagnosis of moderate-to-severe CD. Part A included open-ended questions related to the participant’s overall experiences with CD, fatigue, and impact on daily activities and a cognitive debriefing of several patient-reported outcomes (PROs), including the FACIT-F. Part B consisted of participants completing an electronic daily diary that included the FACIT-F and other PROs for 14 days. Item performance, test–retest reliability, and construct validity were assessed at baseline (Day 1), Day 7, and Day 14.

**Results:**

Thirty-five participants (mean age 45.1 years; 65.7% female) completed an interview (Part A). Ninety-one percent of the interview participants reported fatigue as a symptom attributed to CD. Participants indicated that fatigue had a major impact on their daily activities (e.g., recreation/ hobbies, work/school, yard work and housework), social activities, and emotional health. The FACIT-F was well understood by the interview participants.

Seventy-six participants (mean age 41.9 years; 66% female) completed at least the Day 1 diary entry (Part B). Potential floor and ceiling effects were observed for several FACIT-F items, but test–retest reliability and construct validity were all strong and within the ranges hypothesized a priori.

**Conclusions:**

The interviews indicate that fatigue is a frequent and bothersome symptom experienced by most patients with moderate-to-severe CD and support the content validity of the FACIT-F in this population. Daily diary study results indicate that the FACIT-F scale demonstrates adequate reliability and validity among patients with CD. These study findings suggest that the FACIT-F would be a reliable, valid, and useful measure of fatigue in patients with moderate-to-severe CD.

**Supplementary Information:**

The online version contains supplementary material available at 10.1186/s41687-023-00612-9.

## Background

Crohn’s disease (CD) is a chronic inflammatory disease of the gastrointestinal tract characterized by periods of clinical remission and relapse [1]. CD can affect any part of the gastrointestinal tract but most frequently occurs in the terminal ileum, ileocecal region, colon, and perianal region [2].

Common clinical manifestations of CD include diarrhea, abdominal pain, bowel urgency, weight loss, appetite loss, rectal bleeding, and perianal disease; many patients also suffer from fatigue [3–5]. Patients with CD describe impaired function due to fatigue as one of their most prominent causes of disease-related worry [6]. At the time of CD diagnosis, fatigue prevalence is 48–62% [7].

Prior studies explored the impact of CD on patients’ health-related quality of life (HRQoL) [8–10]. Fatigue reportedly has a detrimental impact on HRQoL and functioning, regardless of disease activity [11–13], and occurs in up to 48% of patients with inflammatory bowel disease (IBD) in clinical remission [14]. Despite its high prevalence, fatigue related to CD is still poorly understood [15].

The Functional Assessment of Chronic Illness Therapy–Fatigue (FACIT-F) scale is a comprehensive set of questions that evaluates different aspects of fatigue in patients suffering with chronic illnesses [16]. It has been validated in the general population [17] as well as in patients with IBD [18] and various other chronic illnesses [17, 19–24]. To date, however, it has not been validated in a population specific to patients with CD. Moreover, the earlier work focused on quantitative validation [18], whereas here, we included qualitative exploration of content validity with patients with moderate to severe CD. It should be noted that establishing content validity is consistent with FDA Guidance and accepted best practices [25].

In this mixed-methods study, we sought to explore the experience of fatigue among patients with moderate-to-severe CD and provide evidence that the FACIT-F has content validity, test–retest reliability, and construct validity in this population. The qualitative interview component (Part A) of the study aimed to confirm the relevance and importance of fatigue to patients with moderate-to-severe CD, cognitively debrief the FACIT-F to establish content validity in this target population, and qualitatively explore meaningful change from the perspective of patients. Secondary objectives of Part A included cognitively debriefing the Overall CD Symptom Patient Global Impression of Severity (PGIS), Overall CD Symptom Patient Global Impression of Change (PGIC), PGIS-Fatigue, and PGIC-Fatigue. The quantitative daily diary study component (Part B) of the study aimed to examine distribution characteristics, test–retest reliability, and construct validity of the FACIT-F.

## Methods

This was a mixed-methods observational study consisting of a cross-sectional qualitative interview (Part A) and a longitudinal quantitative daily diary study (Part B). The study was conducted in compliance with good clinical practice guidelines, including International Conference on Harmonization guidelines [26]. All participants provided informed consent prior to taking part in the study. All study documents were submitted and approved by Advarra Institutional Review Board (IRB) (approval number for Part A: Pro00044594 and approval number for Part B: Pro00046399).

### Part A: Qualitative interview

#### Study design and recruitment

Part A consisted of a single semi-structured, qualitative telephone interview of English-speaking adult patients recruited from six clinical sites aged ≥ 18 years with a clinical diagnosis of moderate-to-severe CD based on laboratory examination of blood and/or stool matter, X-ray, or endoscopic examinations, and experiencing or had previously experienced CD symptoms (e.g., increased number of bowel movements [BMs], BM urgency, abdominal pain) in the past three months, based on self-report. An initial estimated recruitment target of 25 adults was used based on expectations to achieve saturation [27, 28]. A purposive sampling approach was used. Patients who had undergone ileostomy, colostomy, or intra-abdominal surgery in the previous three months were excluded.

#### Qualitative interview

Qualitative interviews were conducted with 35 patients by four interviewers via telephone using a semi-structured interview guide consisting of concept elicitation and cognitive debriefing sections based on literature review results, study research questions, and current knowledge of CD symptoms and impacts. The first portion of the interview guide included open-ended questions related to the participant’s experiences with overall CD and fatigue and their impact on daily activities. Participants completed patient-reported outcomes (PROs) of interest and were subsequently asked questions about the interpretation, clarity, relevance, and feasibility of the individual measures and items. CD remission and meaningful change in CD symptoms, defined as the minimum amount of improvement that would be required for participants to feel that a treatment was worth taking, were also discussed.

This manuscript focuses on fatigue-related PROs, including the FACIT-F and fatigue-specific versions of the PGIS-Fatigue and PGIC-Fatigue. The FACIT-F (version 4; ©1987, 1997) consists of 13 items assessing different aspects of fatigue, with a recall period of the past seven days. A total score is calculated and can range between 0 and 52, with a higher score indicating a lower level of fatigue [16].

The PGIS-Fatigue is a single item assessing the severity of fatigue over the past seven days [29] using a five-level verbal response scale from none (1) to very severe (5). The PGIC-Fatigue is a single item assessing change in fatigue since before the participant entered the study. It also uses a five-level verbal response scale from much better (1) to much worse (5).

The Bowel Urgency Numeric Rating Scale (NRS) is a single-item PRO questionnaire in which respondents rate severity (24-h recall), using a 0 to 10 NRS (0 = “No urgency” and 10 = “Worst possible urgency”).

#### Statistical analysis

Content analyses were conducted using ATLAS.ti (version 8.0 or higher), and qualitative coding and analysis were undertaken by trained researchers [30, 31]. A coding dictionary was developed based on the interview guide and themes and concepts emerging from the interviews. A senior researcher then reviewed each coded transcript for quality control.

Saturation in qualitative research is reached when the inclusion of additional study participants does not provide any substantially new or previously unrecognized concepts [31]. Evidence of saturation in support of PRO instruments to be used as clinical trial endpoints is recommended by the Food and Drug Administration [32]. Interview responses were analyzed to assess novel symptoms observed in each interview. Coding was used to examine the relevance, clarity, and appropriateness of the PRO measures of interest and to assess participant discussion around meaningful change.

Sociodemographic and clinical characteristics were reported using descriptive statistics, including mean, standard deviation (SD), and frequency. Participants’ responses to the PRO measures of interest were summarized using distributional characteristics, including mean, SD, range, floor, and ceiling.

### Part B: Daily diary study

#### Study design and recruitment

Part B consisted of participants completing a web-based survey daily for 2 weeks (14 days). Participants were recruited by a research recruitment vendor utilizing recruiter databases, patient support groups, and/or social media. Eligible participants who completed Part A were also approached to participate.

All participants were screened using an IRB-approved screening script to confirm eligibility. Electronic PRO data were collected electronically using a web-based research survey platform (Baseline Plus) managed by Cisiv.

#### Participant selection

Eligibility criteria mimicked that of Part A, with two key differences: (1) participants’ clinical diagnosis of CD had to be confirmed by either clinical site referral (Part A participants) or an accepted proxy (official summary of a medical appointment or procedure listing the diagnosis, a screen shot of an online health portal or medical records listing the diagnosis, a signed letter from the participant’s clinician confirming the diagnosis, or any other form of evidence deemed acceptable by the study investigator), and (2) participants reporting CD symptoms for < 10 days in the past month (“asymptomatic”) were allowed to participate, up to 20% of the total survey sample. Exclusion criteria were similar to those in Part A but were confirmed based on self-report.

#### Daily diary study

Participants completed the online survey each day for 14 days by logging into Baseline Plus (Cisiv) using their own electronic devices. They were required to respond to all questions at all time points; however, an “opt out” response option was provided for participants who did not want to answer a given question. Each daily survey was only available for 24 h; participants were unable to enter responses for previous days.

The daily survey included Overall CD symptom PGRS, Urgency NRS, and measures of disease severity (i.e.; BM count item and the Abdominal Pain NRS [Table 1]). FACIT-F and PGIS-Fatigue were administered weekly owing to the 7-day recall period inherent in these instruments (Table 1).

**Table 1:** Survey administration schedule

Patient-reported outcome	Number of items	Baseline/ Day 1	Days 2 through 6, 8 though 13	Day 7	Day 14
Sociodemographic and clinical characteristic questionnaire	11	✓			
Overall CD symptom PGRS	1	✓	✓	✓	✓
Overall CD symptom PGIC	1				✓
Abdominal Pain NRS	1	✓	✓	✓	✓
BM count	1	✓	✓	✓	✓
Urgency NRS	1	✓	✓	✓	✓
FACIT-F	13	✓		✓	✓
PGIS-Fatigue	1	✓		✓	✓
PGIC-Fatigue	1				✓
Total items administered at each time point	N/A	65	4	18	20

BM, bowel movement; CD, Crohn’s disease; FACIT-F, Functional Assessment of Chronic Illness Therapy–Fatigue; NRS, numeric rating scale; PGIC, Patient Global Impression of Change; PGIS, Patient Global Impression of Severity; PGRS, Patient Global Rating of Severity

#### Statistical analysis

All analyses were conducted with SAS® statistical software version 9.4 (SAS Institute, Inc.; Cary, NC). No data imputation occurred for missing data, and no multiplicity adjustments were made. All statistical tests were two-tailed and were conducted with Type I error probability fixed at 0.05.

Paired t-tests were used to examine test–retest reliability by comparing the stability of FACIT-F total scores in participants whose response to the PGIS-Fatigue did not change between Day 1 and Day 7 and in those who responded “no change” to the PGIC-Fatigue administered on Day 14. Intraclass correlation coefficient (ICC) > 0.70 and effect size of standardized mean difference (i.e.; Cohen’s d) < 0.20 evaluated stability.

Construct validity of the FACIT-F was examined in relation to PGIS-Fatigue, an overall symptoms Patient Global Rating of Severity (PGRS), BM count, Abdominal Pain NRS, and Bowel Urgency NRS using Spearman correlations at Day 1, Day 7, and Day 14 (with daily diary data were based on the average daily value for days with non-missing values for the Week 1 and Week 2 scores), with the expectation that there would be little difference between scores at Week 1 and Week 2. The Spearman correlations were expected to be moderate to large (r = 0.4–0.7) between the FACIT-F and PGIS-Fatigue, moderate (r = 0.3–0.5) between the FACIT-F and overall CD Symptom PGRS, and small to moderate (r = 0.2–0.5) between the FACIT-F and both BM count and Abdominal Pain NRS. There was no formal a priori hypothesis for the correlation between the FACIT-F and Bowel Urgency NRS, as this exploratory analysis was added after finalization of the statistical analysis plan.

For known-groups validity assessment, i.e., the extent to which an instrument’s scores differentiate between groups of subjects that differ by a relevant clinical (or other) indicator, analysis of variance (ANOVA) models were conducted to assess the significance of Day 7 FACIT-F mean differences. Pairwise differences for FACIT-F at Day 1 were analyzed by PGIS-Fatigue group at Day 1 (none/mild, moderate, severe/very severe) by using post hoc test with Scheffe's method to control for multiple comparisons.

## Results

### Part A: Qualitative interview

#### Participants

Self-reported sociodemographic and clinical characteristics of interview participants are displayed in Table 2. Thirty-five participants were included in Part A. Overall mean (SD) age of participants was 45.1 (15.8) years; 65.7% were female. Thirteen (37.1%) participants had a college degree, while 11 (31.4%) had only finished high school/secondary school. Mean (SD) time since CD diagnosis was 11.9 years for the overall sample. The most common comorbid health conditions reported were hypertension (n = 8; 22.9%), gastroesophageal reflux disease (n = 7; 20.0%), and depression and anxiety (n = 4 each; 11.4%).

**Table 2:** Sociodemographic and clinical characteristics: Part A-qualitative interviews

Characteristic	Total (N = 35)
*Age (years)*	
Mean (SD)	45.1 (15.8)
Median [Range]	46.0 [19–74]
*Gender*, n (%)	
Female	23 (65.7)
*Ethnicity*, n (%)	
Hispanic or Latino	1 (2.9)
Not Hispanic or Latino	34 (97.1)
*Racial background*^a^, n (%)	
American Indian or Alaska Native	1 (2.9)
Asian	1 (2.9)
Black or African American	3 (8.6)
Native Hawaiian or Other Pacific Islander	1 (2.9)
White	28 (80.0)
Other: specified “biracial”	1 (2.9)
Missing	1 (2.9)
*Employment status*, n (%)	
Employed, full-time	17 (48.6)
Employed, part-time	5 (14.3)
Student	3 (8.6)
Retired	5 (14.3)
Disabled	4 (11.4)
Other^b^	2 (5.7)
*Highest level of education*, n (%)	
Secondary/high school	11 (31.4)
Some college	8 (22.9)
College degree	13 (37.1)
Postgraduate degree	2 (5.7)
Other: specified “massage therapist”; STNA	1 (2.9)
*Time since diagnosis* (years)	
Mean (SD)	11.9 (13.7)
Median [Range]	6.8 [0–58]
*Most recent CRP score* (mg/L)	
Mean (SD)	19.3 (32.7)
*Current treatment*^a^, n (%)	
*Biologics*	
Adalimumab	7 (20.0)
Certolizumab	3 (8.6)
Infliximab	3 (8.6)
Ustekinumab	6 (17.1)
Tofacitinib	1 (2.9)
Vedolizumab	4 (11.4)
*Immunomodulators*	
Azathioprine	2 (5.7)
Mercaptopurine	1 (2.9)
*Corticosteroids*	
Budesonide	3 (8.6)
Prednisone	5 (14.3)
*Oral aminosalicylates*	
Sulfasalazine	2 (5.7)
Mesalamine	8 (22.9)
*Anti-diarrheals*	
Loperamide	1 (2.9)
*Comorbid conditions*^a^, n (%)	
No other conditions	13 (37.1)
Allergic rhinitis	3 (8.6)
Anemia	2 (5.7)
Anxiety	4 (11.4)
Arthritis	
*Enteropathic arthritis*	1 (2.9)
Osteoarthritis	3 (8.6)
Rheumatoid arthritis	1 (2.9)
Asthma	3 (8.6)
Cancer^c^	1 (2.9)
COPD	1 (2.9)
Depression	4 (11.4)
GERD	7 (20.0)
Hypertension	8 (22.9)
Multiple sclerosis	1 (2.9)
Psoriasis	1 (2.9)
Other health conditions^a, d^	9 (26.0)

^a^Responses are not mutually exclusive

^b^Specified employment responses (n = 1 each): “self-employed,” “unemployed—but have filed disability and am still battling in court at this time”

^c^History of squamous cell cancer on right calf

^d^Other health conditions reported in n = 1 participant each (not mutually exclusive): acid reflux, kidney stones, osteopenia, gastroesophagitis, irritable bowel syndrome (documented as well-controlled), neuropathy, fibromyalgia, obstructive sleep apnea, colon polyps, osteoporosis, chronic pain syndrome, “sleep disorder”

COPD, chronic obstructive pulmonary disease; CRP, C-reactive protein; GERD, gastroesophageal reflux disease; SD, standard deviation; STNA, state-tested nursing assistant

Most symptom concepts (18/ 28 symptom concepts; 64%) were first endorsed within the first transcript group. Saturation of symptom concepts was reached by the fourth of five transcript groups, with no new symptoms after the 23^rd^ interview.

#### Endorsed symptoms and most bothersome symptoms

During interviews, participants discussed a wide range of symptoms associated with CD. Of the 35 participants, 32 (92%) participants reported experiencing fatigue related to their CD (Additional File 1). This symptom was reported spontaneously by 27 participants (77%) and via probing by five participants (14%). Four patients (13%) considered fatigue to be their most bothersome CD symptom. Other frequently reported symptoms included bowel urgency (n = 34; 97%), abdominal pain or cramping (n = 29; 83%), diarrhea (n = 20; 57%), and increased frequency of BMs (n = 19; 54%).

#### CD remission

Many participants reported that they still expected to have some CD symptoms (n = 21; 60.0%) and/or impacts (n = 17; 48.6%) during remission, but to a lesser degree (i.e., reduced frequency or severity). Five (14.3%) participants specifically cited fatigue as a symptom they could still experience but consider themselves to be in remission.

### Fatigue

#### Experience of fatigue

All participants were asked to share terminology or descriptions of this symptom. Most participants provided multiple terms. The most frequently reported were: “tired” (n = 22), “low energy” or “no energy” (n = 7), “exhausted” or “exhaustion” (n = 6), “weak” (n = 6), “drained” (n = 5), and “worn out” (n = 2) (Additional file 1: Table S1). Other terminology used by one participant each included feeling “sleepy” and having “no strength or stamina.” Supportive quotes provided by participants are shown in Additional file 1: Table S1 and summarized in Table 3.

**Table 3:** Terminology used for “fatigue”

Terminology used^a^	n (%) N = 35	Participant ID, age (years), Supportive quotes
Tired	22 (63)	200–001, age 62*: “You're just, you're just tired. You have no energy.”*
No or low energy	7 (20)	200–010, age 65:* “Low energy. You know, just, you know, being a little lethargic.”*
Exhausted or exhaustion	6 (17)	200–011, age 51: *“Exhausted. That's the word I usually use. I'm exhausted.”*
Weak	6 (17)	600–007, age 20: *“Um, like the weakness—like you're weak, or you're tired.”*
Drained	5 (14)	600–005, age 50: *“Not really, I mean maybe tired-tired, you know; you get drained more. I feel like I’m drained all the time.”*

^a^Terminology is not mutually exclusive; many participants provided multiple terms for fatigue

All participants were asked whether the terms “tiredness,” “lack of energy,” “lack of stamina,” and “sleepiness” were the same or different from the concept of fatigue. Most participants reported that all four terms indicated essentially the same concept as fatigue, with “lack of energy” being most closely related. “Lack of energy” was considered the same or nearly the same concept as fatigue by 32 participants (91%), followed by “tiredness” (n = 30; 86%), “lack of stamina” (n = 29; 83%), and “sleepiness” (n = 28; 80%).

#### Frequency of fatigue

**Fig. 1:**
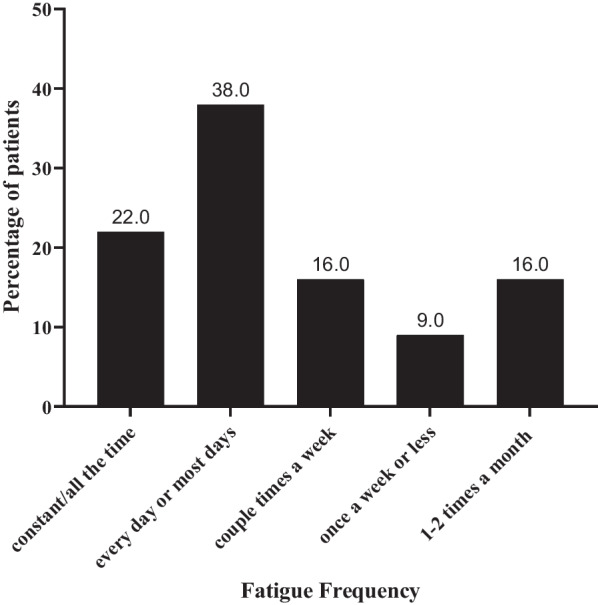
Frequency of fatigue: Part A-qualitative interviews (N = 32). na, not applicable

Figure 1 presents participant-reported fatigue frequency related to CD. Twelve of the 32 participants (38%) reporting CD-related fatigue experienced it approximately every day, while seven (22%) reported constant fatigue. Five participants (16%) said they felt fatigued a couple of times a week, three participants (9%) reported noticing fatigue once a week or less, and five participants (16%) described experiencing fatigue once or twice a month.Three (9%) participants did not report fatigue related to CD and are therefore not included in Fig. 1.

#### Mental versus physical fatigue

While discussing CD-related fatigue, all 35 participants were asked whether they ever experienced mental fatigue. Most participants (n = 26; 74%) reported experiencing both physical fatigue and mental fatigue at times, while six (17%) reported experiencing physical fatigue only. The three participants who reported not having CD-related physical fatigue noted that they do experience mental fatigue, though they did not specify whether this was related to their CD. Participants described mental fatigue as: having difficulty or being unable to “concentrate” or “focus” (n = 15), “being depressed” (n = 5), having a lack of motivation to start or finish things (“not wanting to do stuff,” “can’t do this any longer,” “just done”) (n = 4), and “zoning out” (n = 2). Participants tended to describe physical fatigue as a feeling of not having energy to do something.

#### Impacts of fatigue

The 32 participants reporting CD-related fatigue also described the impacts of fatigue on their lives (Table 4). The most commonly reported impacts were impaired ability to participate in recreation or hobbies, such as exercise and sports (n = 17; 53%); work/school impact (n = 16; 50%); impact on yard- or housework (n = 12; 38%); impact on social activities (n = 8; 25%); causing depression or sadness (n = 8; 25%); general impact on ability to do things (n = 6; 19%); and impact on family life (n = 6; 19%).

**Table 4:** Impacts of fatigue reported by ≥ 2 participants

Impact of fatigue	Total (N = 32)	
	Endorsed n (%)^a^	Most difficult n (%)^b^
Recreation or hobbies (e.g., exercise, sports)	17 (53)	0 (0)
Work/school	16 (50)	9 (56)
Yard- or housework	12 (38)	2 (17)
Social activities	8 (25)	2 (22)
Causes depression or sadness^c^	8 (25)	0 (0)
Family life	6 (19)	3 (43)
Causes frustration or anger^c^	5 (16)	1 (20)
Impacts ability to do things in general	6 (19)	1 (17)
Difficulty concentrating^d^	4 (13)	0 (0)
Confusion^d^	3 (9)	0 (0)
General reduction in QOL	3 (9)	3 (100)
Impacts personal relationships	2 (6)	0 (0)
Staying home	2 (6)	0 (0)
Concerned about future progression of disease^c^	2 (6)	0 (0)

^a^Percentage of participants within that subtype with fatigue who endorsed the impact, within the full sample

^b^Percentage of those endorsing that impact who considered it to be most difficult to deal with, within the full sample

^c^Three participants reported that emotional impacts in general were the most difficult impacts of fatigue

^d^Three participants reported that mental fatigue in general was the most difficult impact of fatigue

n/N, number of participants in group

#### Cognitive debriefing

The distribution of participant responses to each of the 13 items of the FACIT-F for the overall sample is summarized in Additional file 1: Table S2. The full five-point verbal response scale was used, and responses covered the entire range for all but Item 10 (“I am too tired to eat”); no participants used the “very much” response for Item 10.

Mean FACIT-F total scores and mean scores for individual items for the overall sample are shown in Additional file 1: Table S3. The mean (SD) score for the overall sample was 31.3 (13.0).

##### Instructions

Instructions were clear to most participants (n = 29; 83%) with six (17%) recommending minor changes. Recommendations included rewording the instructions (9%: n = 3), providing more examples for the FACIT-F items (3%; n = 1), and changing the recall period (6%; n = 2). Overall thoughts on the FACIT-F and interpretations of its instructions are shown in Additional file 1: Table S4 and issues in Additional file 1: Table S5.

##### Individual items

Participants were asked to discuss each of the 13 FACIT-F items, including item meaning, clarity, and ease of answering.. Overall, FACIT-F items were clear in meaning and easy to answer. Seven (20%) participants had issues understanding Item 3 because the terms “washed out” and “listless” were unfamiliar. Some participants commented that they do not “need” to sleep during the day but would like to, which made interpretation of Item 9 difficult. Select quotations from interview participants regarding interpretations of FACIT-F items are provided (Additional file 1: Table S6).

### Recall of items

Participants were also asked to discuss the appropriateness of the recall period for each of the thirteen FACIT-F items. Overall, participants agreed that the seven-day recall period was appropriate and were able to respond to all items. Relevance of items.

Twenty-one participants indicated that all 13 FACIT-F items were relevant to their experience. Participants were asked to select their top three most and least relevant items: Items 7 (have energy), 4 (tired), and 8 (able to do usual activities) were chosen most often as most relevant (Additional file 1: Fig. S1); Items 10 (too tired to eat), 11 (need help to do usual activities), and 2 (feel weak) were least relevant (Additional file 1: Fig. S2).

#### Interpretation of score change

Participants’ thoughts regarding meaningful change were discussed based upon the top three or four items that would need to change for them to achieve meaningful improvement in their state of fatigue (Additional file 1: Fig. S3). Items 7 and 1 were in the top three for all participants. Participants indicated that a 1- or 2-point change for each of the items that they judged most important could indicate meaningful improvement. Note that this reflects participants’ preliminary interpretation of what a meaningful change could be and is not meant to establish a clinical threshold.

 Additional file 1: Table S7 summarizes cognitive debriefing results for the Overall CD Symptom PGIS, Overall CD Symptom PGIC, PGIS-Fatigue, and PGIC-Fatigue.

### Part B: Daily diary study

#### Participants

Sociodemographic and clinical characteristics of Part B participants are shown (Table 5). Seventy-six participants completed at least the baseline/Day 1 survey entry, and 66 completed all 14 daily surveys. Mean (SD) age of the overall sample was 41.9 (13.2) years, and 66% were female. Mean (SD) time since CD diagnosis was 11.8 (11.8) years. The most frequently self-reported comorbid health conditions were depression (n = 19; 25.0%), hypertension (n = 12; 15.8%), and asthma (n = 8; 10.5%). 29 participants (38.2%) indicated no other known health conditions.

**Table 5:** Sociodemographic and clinical characteristics: Part B-daily diary study

Characteristic	Total N = 76
*Age*	
Mean (SD)	41.9 (13.2)
Median [Range]	40.5 [19–70]
*Gender*, n (%)	
Female	50 (65.8)
*Racial background*, n (%)^a^	
Asian	2 (2.6)
Black or African American	11 (14.5)
White	63 (82.9)
Other (not specified)	1 (1.3)
Missing	1 (1.3)
*Ethnicity*, n (%)	
Hispanic or Latino	4 (5.3)
Not Hispanic or Latino	72 (94.7)
*Employment status*, n (%)^a^	
Employed, full-time	42 (55.3)
Employed, part-time	10 (13.2)
Homemaker/stay-at-home parent	5 (6.6)
Student	5 (6.6)
Unemployed	4 (5.3)
Retired	3 (3.9)
Disabled	9 (11.8)
Other^b^	2 (2.6)
*Highest level of education*, n (%)	
Secondary/high school	4 (5.3)
Some college	12 (15.8)
College degree	46 (60.5)
Postgraduate degree	14 (18.4)
*Time since diagnosis of Crohn’s disease, years*	
Mean (SD)	11.8 (11.8)
Median [Range]	7.5 [0–48]
*Current CD treatment*, n (%)^a^	
I am not currently taking any treatment for my Crohn’s disease	3 (3.9)
Biologic (adalimumab, vedolizumab, ustekinumab, infliximab, golimumab, certolizumab, etc.)	56 (73.7)
Immunomodulators (azathioprine, mercaptopurine, methotrexate, etc.)	17 (22.4)
Corticosteroids (prednisone, budesonide, hydrocortisone, etc.)	14 (18.4)
Other^c^	11 (14.5)
Missing	1 (1.3)
*Comorbid health conditions*, n (%)^a^	
I do not have any other health conditions besides Crohn’s disease	29 (38.2)
Allergic rhinitis	7 (9.2)
Ankylosing spondylitis	1 (1.3)
Anxiety^d^	5 (6.6)
Asthma	8 (10.5)
Celiac disease	3 (3.9)
COPD or emphysema	1 (1.3)
Depression	19 (25.0)
Diabetes (Type 2)	2 (2.6)
Hypertension	12 (15.8)
Multiple sclerosis	1 (1.3)
Psoriasis	5 (6.6)
Osteoarthritis	1 (1.3)
Rheumatoid arthritis	6 (7.9)
Other autoimmune condition(s)^e^	4 (5.3)
Other mental health condition(s)^f^	1 (1.3)
Other health condition(s)^g^	11 (14.5)
Missing	2 (2.6%)

^a^Responses are not mutually exclusive

^b^Other employment status specified by n = 1 each: “short-term disability,” “try to work and do some volunteering”

^c^Other treatment specified by n = 1 each, except where specified otherwise: mesalamine (n = 3), medical marijuana/cannabis (n = 2), “a study medication,” Amitiza, amitriptyline, Lialda, pantoprazole, “Tacro”

^d^Anxiety was not included as a response option in the questionnaire, but as n = 5 participants wrote it in as an “other” response, it is specified in the table

^e^Other autoimmune conditions specified by n = 1, each except where specified otherwise: primary sclerosing cholangitis (n = 2), hidradenitis suppurativa, “liver issues,” prurigo nodularis

^f^Other mental health conditions specified by the same n = 1 participant: PTSD and GAD

^g^Other health conditions specified by n = 1 each, except where specified otherwise: fibromyalgia (n = 2), high cholesterol (n = 2), ankylosis arthritis and scoliosis, fatty liver disease, GERD, Hep B (inactive now) and sleep apnea, hypothyroidism, IIH and POTS, leg lymphedema, Raynaud’s syndrome

Abbreviations: COPD, chronic obstructive pulmonary disease; GAD, generalized anxiety disorder; GERD, gastroesophageal reflux disease; IIH, idiopathic intracranial hypertension; Hep B, Hepatitis B; MS, multiple sclerosis; n/N, number of participants in group; POTS, postural orthostatic tachycardia syndrome; PTSD, posttraumatic stress disorder; SD, standard deviation

#### PRO descriptive characteristics for FACIT-F

FACIT-F total score and individual FACIT-F item scores at Days 1 (n = 76), 7 (n = 64), and 14 (n = 66) are displayed in Table 6. The mean (SD) total scores were 27.6 (12.8), 28.3 (13.8), and 28.4 (13.0), at Days 1, 7, and 14, respectively.

**Table 6:** FACIT-F descriptive characteristics, Days 1, 7, and 14

Measures	Day 1				Day 7				Day 14			
	N	Mean (SD)	Median	Range	N	Mean (SD)	Median	Range	N	Mean (SD)	Median	Range
*FACIT-F*^a^												
Total score^b^	76	27.62 (12.82)	27.0	4.0–50.0	64	28.31 (13.75)	27.5	1.0–51.0	66	28.43 (12.99)	27.0	4.0–50.0
1: I feel fatigued	76	1.24 (1.21)	1.0	0.0–4.0	64	1.44 (1.18)	1.0	0.0–4.0	66	1.55 (1.18)	1.5	0.0–4.0
2: I feel weak all over	76	2.30 (1.21)	2.0	0.0–4.0	64	2.47 (1.36)	2.0	0.0–4.0	66	2.41 (1.24)	2.0	0.0–4.0
3: I feel listless (“washed out”)	76	2.16 (1.35)	2.0	0.0–4.0	64	2.19 (1.32)	2.0	0.0–4.0	66	2.33 (1.32)	2.0	0.0–4.0
4: I feel tired	76	1.28 (1.21)	1.0	0.0–4.0	64	1.45 (1.18)	1.0	0.0–4.0	66	1.50 (1.23)	1.0	0.0–4.0
5: I have trouble starting things because I am tired	76	1.83 (1.41)	2.0	0.0–4.0	64	2.05 (1.45)	2.0	0.0–4.0	66	2.03 (1.38)	2.0	0.0–4.0
6: I have trouble finishing things because I am tired	76	1.96 (1.39)	2.0	0.0–4.0	64	2.17 (1.38)	2.0	0.0–4.0	66	2.03 (1.41)	2.0	0.0–4.0
7: I have energy	76	1.78 (0.92)	2.0	0.0–4.0	64	1.69 (1.02)	1.0	0.0–4.0	66	1.80 (0.93)	2.0	0.0–4.0
8: I am able to do my usual activities	76	2.62 (0.95)	3.0	1.0–4.0	64	2.47 (1.08)	2.0	0.0–4.0	66	2.52 (1.01)	3.0	0.0–4.0
9: I need to sleep during the day	76	2.17 (1.33)	2.0	0.0–4.0	64	2.05 (1.45)	2.0	0.0–4.0	65	2.23 (1.40)	2.0	0.0–4.0
10: I am too tired to eat	76	3.16 (1.06)	4.0	0.0–4.0	64	3.14 (1.15)	4.0	0.0–4.0	66	3.14 (1.12)	3.5	0.0–4.0
11: I need help doing my usual activities	76	3.05 (1.18)	4.0	0.0–4.0	64	3.05 (1.20)	3.0	0.0–4.0	66	2.89 (1.28)	3.0	0.0–4.0
12: I am frustrated by being too tired to do the things I want to do	76	1.93 (1.57)	2.0	0.0–4.0	64	1.91 (1.58)	2.0	0.0–4.0	66	1.74 (1.49)	1.5	0.0–4.0
13: I have to limit my social activity because I am tired	76	2.14 (1.52)	2.0	0.0–4.0	64	2.25 (1.46)	2.0	0.0–4.0	66	2.27 (1.41)	2.0	0.0–4.0

^a^The mean scores for individual FACIT-F items are the mean Likert scale responses; higher scores indicate less severe levels of fatigue

^b^The FACIT-F total score can range from 0 to 52; higher scores indicate less fatigue/better quality of life

Abbreviations: FACIT-F, Functional Assessment of Chronic Illness Therapy–Fatigue; SD, standard deviation

The full range of response options was used for all items except for Item 8 (“usual activities”), for which none of the participants responded, “Not at all” at Day 1. However, the participants did provide the full range of responses for Item 8 at Day 7 and Day 14. For Items 1 (feel fatigued), 4 (feel tired), 5 (trouble starting things), and 12 (frustration from being too tired), high proportions of participants responded “Very much” compared to other response options (27 [35.5%] for Items 1 and 4, 19 [25.0%] for Item 5, and 21 [27.6%] for Item 12) (potential floor effects). For Items 10 (too tired to eat), 11 (need help), 12 (frustration from being too tired), and 13 (social activity), high proportions of participants selected “Not at all” compared to other response options (40 [52.6%], 39 [51.3%], 20 [26.3%], and 20 [26.3%], respectively) (potential ceiling effects).

### Measurement properties of FACIT-F

#### Reliability

The stability and reproducibility of the FACIT-F was assessed using test–retest reliability with participants whose response to the PGIS-Fatigue did not change between Day 1 and Day 7 (n = 43) and with those with a PGIC-Fatigue response of “no change” at Day 14 (n = 35). Between Days 1 and 7 (using PGIS-Fatigue), the ICC for the overall sample was 0.92, which is indicative of excellent test–retest reliability; the effect size was 0.07, which is indicative of instrument stability (Table 7). At Day 14 (using PGIC-Fatigue), the ICC was also very large (0.94), while the effect size was very low (0.01), indicating that the FACIT-F is a stable instrument (Table 8).

**Table 7:** FACIT-F test–retest reliability, Days 1 and 7, based on PGIS-fatigue

FACIT-F^a^	N	Day 1 mean (SD)	Day 7 mean (SD)	Difference score^b^	t-value	*p* value^c^	ES	ICC
Total score—overall sample	43	30.07 (13.51)	31.05 (14.12)	0.98	1.14	0.2595	0.07	0.92

^a^Among patients who had same PGIS-Fatigue scores at Day 1 and Day 7

^b^Calculated as the Day 7 mean score minus the Day 1 mean score

^c^Paired t-tests comparing responses at Day 1 and Day 7

ES, effect size; FACIT-F, Functional Assessment of Chronic Illness Therapy–Fatigue; ICC, intraclass correlation coefficient; PGIS, Patient Global Impression of Severity; SD, standard deviation

**Table 8:** FACIT-F test–retest reliability, Days 7 and 14, based on no change in PGIC-fatigue

FACIT-F^a^	N	Day 7 mean (SD)	Day 14 mean (SD)	Difference score^b^	t-value	*p* value^c^	ES	ICC
Total score—overall sample	35	29.31 (13.92)	29.19 (13.97)	− 0.13	− 0.15	0.8780	0.01	0.94

^a^Among patients defined as having no change in fatigue as measured by the overall CD symptom PGIC-Fatigue (at Day 14)

^b^Calculated as the Week 2 mean score minus the Week 1 mean score

^c^Paired t-tests comparing responses at Day 7 and Day 14

ES, effect size; FACIT-F, Functional Assessment of Chronic Illness Therapy–Fatigue; ICC, intraclass correlation coefficient; PGIC, Patient Global Impression of Change; SD, standard deviation

#### Construct validity

Construct validity of FACIT-F was assessed versus PGIS-Fatigue at Days 1, 7, and 14. Strong Spearman correlation coefficients (ranging from − 0.81 to − 0.84) were observed at each time point and larger than moderate-to-large hypothesized correlations (Table 9). All correlations were statistically significant, supporting validity of the measure. Construct validity was also assessed against the Overall CD Symptom PGRS, the BM count, and the Abdominal Pain NRS. Large correlations were observed between the FACIT-F and Abdominal Pain NRS (ranging from − 0.51 to − 0.66), moderate to large correlations were observed between the FACIT-F and the overall CD symptom PGRS (ranging from − 0.46 to − 0.58), and moderate correlations were observed between FACIT-F and the BM count (ranging from − 0.38 to − 0.40). Correlations at Week 1/Day 7 were larger in magnitude than correlations at Week 2/Day 14. However, correlations were either within (overall CD symptom PGRS and BM count) or larger than (Abdominal Pain NRS) the hypothesized ranges. Lastly, construct validity was assessed against the Bowel Urgency NRS. Correlations were moderate at both time points but were larger in magnitude at Day 14/Week 2 (− 0.53) than at Day 7/Week 1 (− 0.45).

**Table 9:** FACIT-F construct validity: PGIS-fatigue, overall CD symptom PGRS, BM count, abdominal pain NRS, and bowel urgency NRS

	PGIS-Fatigue			Overall CD PGRS			BM count			Abdominal Pain NRS			Bowel Urgency NRS		
	Corr	N	*p* value	Corr	N	*p* value	Corr	N	*p* value	Corr	N	*p* value	Corr	N	*p* value
Day 1	− 0.81	76	< 0.0001	NA	NA	NA	NA	NA	NA	NA	NA	NA	NA	NA	NA
Day 7	− 0.84	64	< 0.0001	− 0.58	64	< 0.0001	− 0.40	63	0.0011	− 0.66	64	< 0.0001	− 0.45	64	0.0002
Day 14	− 0.82	66	< 0.0001	− 0.46	66	0.0001	− 0.38	65	0.0017	− 0.51	66	< 0.0001	− 0.53	66	< 0.0001

CD, Crohn’s disease; Corr, correlation; FACIT-F, Functional Assessment of Chronic Illness Therapy–Fatigue; NA, not available; NRS, numeric rating scale; PGIS, Patient Global Impression of Severity; PGRS, Patient Global Rating of Severity

#### Known-groups validity of FACIT-F

Known-group calculations for the FACIT-F using PGIS-Fatigue scores are shown in Table 10. On Day 1, mean FACIT-F score decreased as the mean PGIS-Fatigue score increased (both indicating more severe fatigue), and all pairwise comparisons had a statistically significant difference (≤ 0.002). Known-group calculations were consistent, and pairwise comparisons were statistically significant when Day 7 FACIT-F data and Week 1 mean scores from the overall CD symptom PGRS were used (Table 11).

**Table 10:** Known-groups validity of the FACIT-F, Day 1

Anchor	FACIT-F Day 1 score		Overall F-test		Pairwise comparison^a^ (*p* value)
	N	Mean (SD)	Test value	*p* value	
PGIS-Fatigue Day 1 mean scores			82.9	< 0.0001	1: < 0.0001, 2: < 0.0001, 3: 0.0002
None/mild	21	43.8 (3.9)			
Moderate	33	24.9 (8.8)			
Severe/very severe	22	16.2 (6.9)			

^a^Pairwise comparisons between means were performed using Scheffe’s test, adjusting for multiple comparisons:

1 = None/mild versus moderate

2 = None/mild versus severe/very severe

3 = Moderate versus severe/very severe

FACIT-F, Functional Assessment of Chronic Illness Therapy–Fatigue; PGIS, Patient Global Impression of Severity; SD, standard deviation

**Table 11:** Known-groups validity of the FACIT-F, Day 7

Anchor	FACIT-F Day 7 score		Overall F-test	
	N	Mean (SD)	Test value	*p* value
Overall CD PGRS Week 1 mean scores			17.94	< 0.0001
< Median	33	34.58 (14.3)		
≥ Median	31	21.65 (9.48)		

FACIT-F, Functional Assessment of Chronic Illness Therapy–Fatigue; PGRS, Patient Global Rating of Severity; SD, standard deviation

## Discussion

Qualitative interview findings demonstrated that fatigue is an important symptom among individuals with moderate to severe CD. Physical and mental fatigue was reported by most participants. Participants reported a lack of/low energy and feeling “weak,” “drained,” “exhausted,” and “worn out”. They described fatigue that could be constant or occur several times a week and that interfered with their daily activities, social life, and emotional health. The mean FACIT-F scores from the qualitative interview and daily diary samples were much lower than the estimated mean (SD) scores of 43.6 (9.4) for the US general population [17], indicating that participants experienced more severe fatigue than the general population. Thus, fatigue is an important symptom to assess. These findings are consistent with previous studies of fatigue in patients with IBD [7, 18, 33].

The qualitative interview results confirm that in patients with moderate-to-severe CD, fatigue negatively impacts HRQoL, which is concordant with previous studies in patients with IBD and other chronic illnesses [13, 34]. Moreover, patients with CD can still experience fatigue during remission, as reported in previous studies (14, 15). The qualitative interviews show that fatigue is a frequent, bothersome, and clinically important symptom experienced by most patients with CD and support the content validity of the FACIT-F in a CD population.

Qualitative interview results also indicated that the FACIT-F was well understood overall. The instructions were clear to most participants, and the FACIT-F items were clear in meaning and easy to answer. However, a few minor problems with comprehension and relevance of the FACIT-F were reported. Some participants thought the recall period was too long for individual items, while others thought seven days was too short to reflect their experiences. Participants also reported difficulties responding to certain items because their symptoms varied over the seven-day recall period and suggested ways in which certain items could be reworded for added clarity. Items 7 (“I have energy”), 4 (“I feel tired”), and 8 (“I am able to do my usual activities”) were chosen most often as being most relevant, and Items 10 (“I am too tired to eat”), 11 (“I need help doing my usual activities”), and 2 (“I feel weak all over”) were commonly chosen as being least relevant.

The FACIT-F scale has been validated in an IBD patient population [18]; however, the study in which it was validated utilized physician assessments (i.e., Physician’s Global Assessment of patient’s health status and Harvey-Bradshaw Index) and biomarkers (erythrocyte sedimentation rate and C-reactive protein) as anchors for psychometric evaluations. Although our study used other PROs as anchors, test–retest reliability remained very strong, and validity was also demonstrated. As anticipated, the largest correlations were with the PGIS-Fatigue (> − 0.81 at all three time points), while large correlations were observed between the FACIT-F and Abdominal Pain NRS, moderate to large between the FACIT-F and the overall CD symptom PGRS, and moderate correlations between FACIT-F and BM count as well as the Bowel Urgency NRS. These preliminary results from our pilot study are integral in demonstrating that the FACIT-F scale works well in this patient population and supporting its use and further exploration as an endpoint in future studies and clinical trials.

Participants frequently reported that they expect to continue to have symptoms, including fatigue, even when their disease is in remission. This may be a result of adaptation to chronic illness. Investigators considering the use of symptom outcomes, such as fatigue, in clinical trials need to consider this, as the ability to improve fatigue in patients who consider themselves to be in remission may be very different than in those who consider themselves to have active disease.

Potential floor and ceiling effects must be interpreted with caution given the relatively small sample size of this study. Of note, the FACIT-F questionnaire was originally developed for patients with cancer and anemia, patient populations with a potentially higher level of fatigue than patients with CD. The ceiling effect for Item 13 (limited social activities) could also be explained by the recent COVID-19 pandemic. However, given that the full five-point verbal response scale was used for all but Item 8 at Day 1 and that Items 7, 4, and 8 were frequently selected by the patients as the most relevant items of the questionnaire, these items still seem relevant for patients with CD, especially those with active moderate-to-severe CD.

Part A of this study was qualitative and employed purposive sampling to recruit participants. Although recruiting a diverse sample was the goal, the study was limited by available patient pools and recruitment timelines and may not be representative of the greater CD population in the US or globally. Similarly, since approximately 80% of daily diary study participants in Part B of the study were recruited via a research recruitment vendor, the sample may not be representative of other patients with CD in the US. Lastly, while daily diary study participants recruited through the research recruitment vendor were required to provide evidence of CD diagnosis, no clinical data were available to clinically define or confirm disease severity among these participants.

An important limitation of this study is that depression is a potential confounder for the results. We do not know the full scope of depression among the study sample for Part B, in particular, as rates of depression were based on self-report. While participants were asked whether they attributed their fatigue to CD, it is still possible that fatigue could also have been due to depression, other comorbidities, or medications that the participants were taking. Another limitation, common to qualitative research, particularly with smaller sample sizes [35], is the size of available patient pools and recruitment timelines, so that the sample might not be representative of the greater CD population. The possibility exists that survey (Part B) participants may not be representative of other CD patients in the US, as most survey participants were recruited via a research recruitment vendor. In addition, clinical data were not available to clinically define or confirm disease severity, although CD diagnosis was confirmed for each participant. Many participants were unaware of their CD subtype, and we were unable to verify responses for those who did report a subtype.

## Conclusion

In summary, results from the qualitative portion of this study indicate that fatigue is an important symptom concept in patients with CD. Daily diary study results indicate that the FACIT-F scale demonstrates adequate reliability and validity among patients with CD. Overall, study results reported here support the use of the FACIT-F scale in studies of patients with CD that include further exploration of meaningful change and responsiveness using clinical trial data.

## Supplementary Information

**Additional file 1: Table S1.** Terminology used for “Fatigue”. **Table S2.** Cognitive debriefing: Summary of item-level discussion for the FACIT-F.** Table S3.** Mean FACIT-F scores: Part A-qualitative.** Table S4.** Overall Thoughts on the FACIT-F and Interpretation of FACIT-F Instructions.** Table S5.** Reported issues with FACIT-F instructions.** Table S6.** Participant interpretation of individual FACIT-F items.** Table S7.** Cognitive debriefing results for overall CD symptom PGRS, overall CD symptom PGIC, PGIS-Fatigue, and PGIC-Fatigue.** Figure S1.** Most relevant FACIT-F items: Part A-qualitative interviews.** Figure S2.** Least relevant FACIT-F items: Part A-qualitative interviews.** Figure S3.** Most important FACIT-F items for improvement: Part A-qualitative interviews.

## Data Availability

The datasets used and analyzed for the current study are available from the corresponding author upon reasonable request.

## Abbreviations

CD: Crohn’s disease
FACIT-F: Functional Assessment of Chronic Illness Therapy–Fatigue
HRQoL: Health-related quality of life
IBD: Inflammatory bowel disease
ICC: Intraclass correlation coefficient
IRB: Institutional review board
NRS: Numeric rating scale
PGIC: Patient Global Impression of Change
PGIS: Patient Global Impression of Severity–Fatigue
PRO: Patient-reported outcome
SD: Standard deviation
