# Supplementary material for: Experience and measurement of fatigue in adults with Crohn’s disease: results from qualitative interviews and a longitudinal 2-week daily diary pilot study
Source: J Patient Rep Outcomes. 2023 Jul 20;7:75. doi: 10.1186/s41687-023-00612-9 (PMC10359232; doi:10.1186/s41687-023-00612-9)
Supplement: Supplementary file 1 — Additional file 1: Table S1. Terminology used for “Fatigue”. Table S2. Cognitive debriefing: Summary of item-level discussion for the FACIT-F. Table S3. Mean FACIT-F scores: Part A-qualitative. Table S4. Overall Thoughts on the FACIT-F and Interpretation of FACIT-F Instructions. Table S5. Reported issues with FACIT-F instructions. Table S6. Participant interpretation of individual FACIT-F items. Table S7. Cognitive debriefing results for overall CD symptom PGRS, overall CD symptom PGIC, PGIS-Fatigue, and PGIC-Fatigue. Figure S1. Most relevant FACIT-F items: Part A-qualitative interviews. Figure S2. Least relevant FACIT-F items: Part A-qualitative interviews. Figure S3. Most important FACIT-F items for improvement: Part A-qualitative interviews. [file 41687_2023_612_MOESM1_ESM.docx]

**Online Supplement**

| Supplemental Table 1. Terminology Used for “Fatigue” | | |
| --- | --- | --- |
| Terminology used ^a^ | n (%); N = 35 | Participant ID, age (years), Supportive quotes |
| Tired | 22 (63) | 200-007, age 36*: “Um, very—I'm very tired. Um, I could sleep, uh, eight hours. And I, I don’t normally sleep eight hours, but I could sleep eight hours and still feel completely exhausted.”*  200-001, age 62*: “You're just, you're just tired. You have no energy.”*  600-004, age 66: *“Guess just being tired all the time…I just don’t think that I can go on and keep going, I'm so tired. I just don’t have it in me to, um, do that today or, or go on today. I need to, I need to lie down.”*  600-005, age 50: “*It’s almost like if you’ve been working out so much and then all of a sudden, you just like drop because you can’t do anymore, you know, if you’re out walking or what have you. You’ve been walking all day or something, and then you’re like, ‘Oh God, I’ve got to stop because I’m tired’. That's kind of a constant thing with me; it’s not just a little bit here and a little bit there; it’s all the time. I’m tired all the time.”* |
| No or low energy | 7 (20) | 200-004, age 70: *“…the fatigue to me just, I just don’t have the energy. I just—it makes me want to—I could sit down and fall asleep. I could go to bed and sleep. And I have days like that to where I get really worn out to where I just, I just want to sleep. I just don’t want to be bothered.”*  200-010, age 65: *“Low energy. You know, just, you know, being a little lethargic.”*  700-003, age 31: *“Fatigue means like you don’t have enough—like you're down. Your energy, your energy level is down. Your body can't keep up, but what—or you're, or you're not consuming enough water or you're not eating enough.”* |
| Exhausted or exhaustion | 6 (17) | 600-008, age 31: *“Just constant exhaustion; it’s really hard to explain it beyond like it’s like a tiredness where you will wake up and you just feel like you spent all day busy and working hard, and you wake up with that exhaustion like you’ve done a hard day’s work, but you haven't.”*  200-011, age 51: *“Exhausted. That's the word I usually use. I'm exhausted.”*  200-007, age 31: *“Um, very—I'm very tired. Um, I could sleep, uh, eight hours. And I, I don’t normally sleep eight hours, but I could sleep eight hours and still feel completely exhausted.”* |
| Weak | 6 (17) | 600-007, age 20: *“Um, like the weakness—like you're weak, or you're tired.”*  200-002, age 34: “*Um, weak, um, tired, um, I'm trying to think. Um, weak and tired, definitely.”*  700-001, age 45*: “Just being physically weak from not eating.”* |
| Drained | 5 (14) | 600-005, age 50: *“Not really, I mean maybe tired-tired, you know; you get drained more. I feel like I’m drained all the time.”*  300-005, age 52: *“You get so tired to the point where you’re just like, ‘I can’t do this no more, I’m so tired, Lord, I can’t do this no more’. You’re just drained; you’re so drained.”*  200-012, age 56: *“Just tired, lack of energy, feel drained; that would be the words that I would use.”*  100-004, age 20: “*I would describe it I guess as…you wanna sleep more and…you're always just tired. Like, you—you just always feel drained of energy.”* |
| ^a^ Terminology is not mutually exclusive; many participants provided multiple terms for fatigue. | | |

| Supplemental Table 2. Cognitive Debriefing: Summary of Item-Level Discussion for the FACIT-F | | | |
| --- | --- | --- | --- |
|  | Yes  n (%) | No  n (%) | Missing or response unclear  n (%) |
| Instructions | | | |
| Understood as intended^a^ | 34 (97) | 1 (3) | 0 (0) |
| Structure of instrument is clear/reasonable | 34 (97) | 1 (3) | 0 (0) |
| Item 1 (feel fatigued) | | | |
| Understood as intended^a^ | 35 (100) | 0 (0) | 0 (0) |
| Relevant concept^b^ | 35 (100) | 0 (0) | 0 (0) |
| Suitable recall period^c^ | 32 (91) | 1 (3) | 2 (6) |
| *Indicated thinking about a shorter period (“from when I woke up until now”): 1 (3)* | | | |
| Suitable scale/response options^d^ | 32 (91) | 0 (0) | 3 (9) |
| Item 2 (feel weak all over) | | | |
| Understood as intended^a^ | 34 (97) | 1 (3) | 0 (0) |
| Relevant concept^b^ | 31 (89) | 3 (9) | 1 (3) |
| Suitable recall period^c^ | 25 (71) | 7 (20) | 3 (9) |
| *Indicated thinking about a shorter period (“from when I woke up until now”): 1 (3)* | | | |
| *Recall period should be shorter (e.g., last three to four days): 5 (14)* | | | |
| *Recall period should be longer (a month or two): 1 (3)* | | | |
| Suitable scale/response options^d^ | 30 (86) | 0 (0) | 5 (14) |
| Item 3 (feel listless [“washed out”]) | | | |
| Understood as intended^a^ | 28 (80) | 7 (20) | 0 (0) |
| Relevant concept^b^ | 28 (80) | 0 (0) | 7 (20) |
| Suitable recall period^c^ | 26 (74) | 4 (11) | 5 (14) |
| *Recall period should be shorter (e.g., last two to three days): 4 (11)* | | | |
| Suitable scale/response options^d^ | 26 (74) | 4 (11) | 5 (14) |
| Item 4 (feel tired) | | | |
| Understood as intended^a^ | 35 (100) | 0 (0) | 0 (0) |
| Relevant concept^b^ | 35 (100) | 0 (0) | 0 (0) |
| Suitable recall period^c^ | 24 (69) | 4 (11) | 7 (20) |
| *Recall period should be shorter (e.g., last two to three days): 4 (11)* | | | |
| Suitable scale/response options^d^ | 30 (86) | 0 (0) | 5 (14) |
| Item 5 (trouble starting things) | | | |
| Understood as intended^a^ | 35 (100) | 0 (0) | 0 (0) |
| Relevant concept^b^ | 33 (94) | 2 (6) | 0 (0) |
| Suitable recall period^c^ | 22 (63) | 4 (11) | 9 (26) |
| *Recall period should be shorter (unspecified length): 3 (9)* | | | |
| *Recall period should be longer (past couple of weeks or past month): 1 (3)* | | | |
| Suitable scale/response options^d^ | 30 (86) | 1 (3) | 4 (11) |
| Item 6 (trouble finishing things) | | | |
| Understood as intended^a^ | 35 (100) | 0 (0) | 0 (0) |
| Relevant concept^b^ | 33 (94) | 2 (6) | 0 (0) |
| Suitable recall period^c^ | 21 (60) | 4 (11) | 10 (29) |
| *Recall period should be shorter (unspecified length): 3 (9)* | | | |
| *Recall period should be longer (past couple of weeks or past month): 1 (3)* | | | |
| Suitable scale/response options^d^ | 29 (83) | 1 (3) | 5 (14) |
| Item 7 (have energy) | | | |
| Understood as intended^a^ | 33 (94) | 2 (6) | 0 (0) |
| Relevant concept^b^ | 33 (94) | 0 (0) | 2 (6) |
| Suitable recall period^c^ | 21 (60) | 3 (9) | 11 (31) |
| *Recall period should be shorter (unspecified length): 1 (3)* | | | |
| *Recall period should be longer (e.g., 14 days): 2 (6)* | | | |
| Suitable scale/response options^d^ | 29 (83) | 2 (6) | 4 (11) |
| Item 8 (able to do usual activities) | | | |
| Understood as intended^a^ | 35 (100) | 0 (0) | 0 (0) |
| Relevant concept^b^ | 34 (97) | 1 (3) | 0 (0) |
| Suitable recall period^c^ | 19 (54) | 3 (9) | 13 (37) |
| *Recall period should be shorter (unspecified length): 3 (9)* | | | |
| Suitable scale/response options^d^ | 28 (80) | 3 (9) | 4 (11) |
| Item 9 (need to sleep during the day) | | | |
| Understood as intended^a^ | 34 (97) | 1 (3) | 0 (0) |
| Relevant concept^b^ | 32 (91) | 2 (6) | 1 (3) |
| Suitable recall period^c^ | 21 (60) | 3 (9) | 11 (31) |
| *Recall period should be shorter (unspecified length): 3 (9)* | | | |
| Suitable scale/response options^d^ | 29 (83) | 0 (0) | 6 (17) |
| Item 10 (too tired to eat) | | | |
| Understood as intended^a^ | 34 (97) | 1 (3) | 0 (0) |
| Relevant concept^b^ | 23 (67) | 11 (31) | 1 (3) |
| Suitable recall period^c^ | 21 (60) | 2 (6) | 12 (34) |
| *Recall period should be shorter (unspecified length): 2 (6)* | | | |
| Suitable scale/response options^d^ | 27 (77) | 1 (3) | 7 (20) |
| Item 11 (need help doing usual activities) | | | |
| Understood as intended^a^ | 35 (100) | 0 (0) | 0 (0) |
| Relevant concept^b^ | 29 (83) | 6 (18) | 0 (0) |
| Suitable recall period^c^ | 21 (60) | 2 (6) | 12 (34) |
| *Recall period should be shorter (unspecified length): 2 (6)* | | | |
| Suitable scale/response options^d^ | 29 (83) | 0 (0) | 6 (18) |
| Item 12 (frustrated by being too tired) | | | |
| Understood as intended^a^ | 34 (97) | 0 (0) | 1 (3) |
| Relevant concept^b^ | 34 (97) | 1 (3) | 0 (0) |
| Suitable recall period^c^ | 21 (60) | 2 (6) | 12 (34) |
| *Recall period should be shorter (unspecified length): 2 (6)* | | | |
| Suitable scale/response options^d^ | 27 (77) | 1 (3) | 7 (20) |
| Item 13 (limit social activity) | | | |
| Understood as intended^a^ | 35 (100) | 0 (0) | 0 (0) |
| Relevant concept^b^ | 34 (97) | 1 (3) | 0 (0) |
| Suitable recall period^c^ | 21 (60) | 2 (6) | 12 (34) |
| *Recall period should be shorter (unspecified length): 2 (6)* | | | |
| Suitable scale/response options^d^ | 27 (77) | 0 (0) | 8 (23) |
| ^a^ Instructions or items were “understood as intended” when no issues were raised and the participants’ explanation of how they thought about the item was logical/correct.  ^b^ Participants were not asked to indicate for each individual item whether it was relevant or not. All 35 participants were instead asked, overall, which FACIT-Fatigue items were most relevant to their experience and which (if any) were not relevant to their experience. In this table, the “Relevant Concept” row summarizes this discussion. Participants who indicated that an item was “not relevant” are counted in the “No” column, and everyone else is counted in the “Yes” column. Participants who did not seem to understand the intended meaning of an item when discussing each individual item in turn are counted in the “Missing” column for relevance.  ^c^ Due to lack of time and interview fatigue resulting from the repetitive nature of the probing, not all participants were asked to comment on the recall period for every individual item. The “Suitable Recall Period” therefore includes a lot of participants in the “Missing or Response Unclear” column. These participants did not indicate there were any issues with the recall period, but they did not explicitly say the recall period was appropriate.  ^d^ Due to lack of time and interview fatigue resulting from the repetitive nature of the probing, not all participants were asked to comment on the response scale for each individual item. The response scale was deemed suitable when participants indicated they did not have any issues choosing a response. If participants did not comment on responding, they were counted as “missing” in the table.  Abbreviations: FACIT-F, Functional Assessment of Chronic Illness Therapy–Fatigue | | | |

| Supplemental Table 3. Mean FACIT-F Scores: Part A-Qualitative Interviews | |
| --- | --- |
| FACIT-F Response | Total  N = 35 |
| Mean total score (SD) | 31.3 (12.95) |
| Item 1 (feel fatigued) | 1.9 (1.30) |
| Item 2 (weak all over) | 2.6 (1.24) |
| Item 3 (listless/washed out) | 2.6 (1.40) |
| Item 4 (feel tired) | 1.6 (1.24) |
| Item 5 (trouble starting things) | 2.2 (1.42) |
| Item 6 (trouble finishing things) | 2.2 (1.33) |
| Item 7 (have energy) | 2.1 (1.12) |
| Item 8 (usual activities) | 2.3 (1.16) |
| Item 9 (sleep during the day) | 2.5 (1.12) |
| Item 10 (too tired to eat) | 3.4 (0.95) |
| Item 11 (need help) | 3.2 (1.15) |
| Item 12 (frustration from being too tired) | 2.2 (1.60) |
| Item 13 (limited social activity) | 2.4 (1.40) |
| Abbreviations: FACIT-F, Functional Assessment of Chronic Illness Therapy–Fatigue; SD, standard deviation | |

| **Supplemental Table 4. Overall Thoughts on the FACIT-F and Interpretation of FACIT-F Instructions** | |
| --- | --- |
|  | **Supportive quotes** |
| Thoughts about the recall period | 200-001, age 61: *“In the last seven days. I like the last seven days, because that's good to limit it…We have to limit, because, yeah, if we think about the last, you know, forever, yeah, good to have limits. Yeah, good to narrow my—yeah, because my brain is a very scary place. Good to narrow me.”*  **INTERVIEWER: “Okay, and then, what time period were you asked to consider for the, sorry, what time period you asked to consider, from when to when?”** 300-004, 41, Type 1: “*The past 7 days so since last Friday to this Friday.”*  600-008, age 31: *“Roughly the last 7 days to 9 days, because like even I’m thinking back to this past Tuesday and stuff because even that's relatively fresh.”*  **INTERVIEWER: “*And what time period were you asked to consider?”*** 700-007, 46, Type 4: *“The last seven days. Like, the last week.”* |
| Thoughts about the structure of the instrument and response scale | **INTERVIEWER: *“what did you think about the way the questions were structured, structured kind of in that like table/grid format? Is that—is it clear how you're supposed to answer?”*** 200-001, 61: *“Yeah. I like this better…Gives you less choices. Zero to four is way easier than zero to ten to me.”*  200-007, age 36: *“…once I realized what the first thing wanted me to do, each item was, was very well written out to where I knew what to answer.”*  **INTERVIEWER: *“And then what do you think about the way the questions are structured? Is it clear how you're supposed to answer each one?”*** 300-002, age 22: *“Uh, yeah. Everything was perfectly phrased. I understood it with ease.”*  100-002, age 69: *“I've seen surveys where they say, um, never, sometimes, often, and always. And I think that the never and always have always been a problem for me. But the way you worded it, not at all and very much, I think was a really good way to do it… I think, I think it just makes it easier for people to understand what you're looking for.”*  700-001, age 44: *“if you want someone to think about the past seven days, you can't phrase the question in the present moment…Like when you say, I have, when you say, I have energy, the first thing that comes to mind is, no, I don’t right in this moment, but, you know, I have had energy if you tell someone to think over the past seven days, it digs a little bit deeper to, you know, the past week.”* |
| Interpretation and thoughts about the instructions | 200-001, age 61: *“So it's asking me in the past week, of all these questions that you're asking, to go over and say how you feel. Not at all, a little bit, somewhat, quite a bit, or very much…Circle them.”*  200-004, age 70: *“It's basically asking me how, myself, how I feel about these items. Um, and that's the way I'm reading it. It looks like other people have said that these are things that I guess must be important questions that everybody needs to ask for theirselves* [sic] *I guess. I guess other people this is how things that have bothered them, so I guess they're trying to figure out how this bothers you.”*  700-001, age 44: *“Um, over the last week, it's asking me how fatigued I've been, what issues it's caused me, and if I wasn’t, what could I do or what would I do basically.”*  200-008, age 58: *“Uh, they're asking me to rate, uh, my level of fatigue as it relates to activities, uh, in my daily functioning.”*  **INTERVIEWER: *“Are these instructions clear in meaning or are there any changes that you would recommend to make them clearer?”*** 300-002, age 21: *“Uh, no. This was perfectly phrased.”* |

Abbreviations: FACIT-F, Functional Assessment of Chronic Illness Therapy–Fatigue

| **Supplemental Table 5. Reported Issues with FACIT-F Instructions** | |
| --- | --- |
| **Recommendation** | **Supportive quotes** |
| Rewording instructions | **INTERVIEWER:** “***And then, were the instructions clear in meaning, or are there any changes that you would recommend to make them clearer?”*** 100-004, age 19: “*I mean, maybe it could be worded differently. Like when it says below is a list of statements that other people with your illness have said are important, and it—and then it says please select one number per line to indicate response. It doesn't really say, I guess, what I'm responding about. So, maybe talk about how—how like related to these—to like, um, maybe my symptoms or something and like how much like—like I can agree with.”*  200-007, age 26: *“It just threw me off when it said, uh, like the first thing says like below is a list of statements that other people with your illness have said are important. So it made me think, um, that like this is their importance of like how often they wouldn’t want to feel that. Like, for instance, the I feel fatigued, I thought they wanted me to answer it like no, not at all. Like I don’t want to feel fatigued…Not that, do I feel fatigued.”*  200-006, age 29: *“I think* [the instructions are] *clear. Um, I would recommend removing that first sentence, which is, uh, below is a list of statements that other people with your illness have said are important. Um, I just feel like that's a little bit confusing, and it kind of detracts from, uh, from just, uh, reading the directions there, which says please select one number per line to indicate your responses.”* |
| Providing examples for individual items in general | 100-003, age 23: *“Um, I guess giving, giving a few examples. Um, for Question 3, there's an example of ‘washed out’. I mean, for some people, they probably will have different interpretations of what these mean. Like ‘I have energy’. What does that mean? Do you do any chores, or do I have energy to go, go for a jog? Like, you know, it's kind of probably distinguish between different activities.”* |
| Changing recall period | 200-005, age 61*: “I think a day to day thing would be more accurate for you because even feeling great the last seven days, there are some episodes where I didn’t feel quite as great.”*  **INTERVIEWER: *“And do you think that time period is appropriate?”*** 200-008, 58*: “No. I, I would say the past three months. That's what I would say….”* **INTERVIEWER*: For all of them?*** 200-008: *“Yeah. Pretty much… It really should say, how do you feel for—it applies to the past three months, because I've been in remission, you know, for a long time.”* |

Abbreviations: FACIT-F, Functional Assessment of Chronic Illness Therapy–Fatigue

**Supplemental Table 6. Participant Interpretation of Individual FACIT-F Items**

| **Items** | **Supportive quotes** |
| --- | --- |
| Item 1 (feel fatigued) | 200-007, age 36: *“The ‘I feel fatigued’ is the like overall, my overall like everything. Um, not just, you know, do I feel tired, like I need to take a nap, but like mentally, physically, like that's what I take as fatigued.”*  700-004, age 50: *“I see fatigue as a different thing as being tired. So, um, just like that heavy, exhausted feeling.”* |
| Item 2 (weak all over) | 100-004, age 19: *“It's just asking like how like—do you feel like you're weak, like weakness or like—like fragile.*  200-001, age 61*: “To me that's asking if, um—weak all over just feels like a whole, um—that almost to me implies—weak all over just is like unable to function almost mentally and physically.”* |
| Item 3 (listless/washed out) | 200-004, age 70: *“Well, being washed out, that's—I have those days to where I just don’t want to do anything. That these are my days that I just lie in bed, or I could just sit in a chair and sleep all day or just stay there.”*  600-004, age 66: *“You're just done. You, um, you're wiped out, tired; don’t ask me to get up and do anything.”* |
| Item 4 (feel tired) | 700-002, age 28: *“I thought that was kind of self-explanatory, being tired, just like how sleepy I am.”*  100-001, age 56: *“I just thought about the past week if I—you know, how I've been feeling, if I have been tired.”* |
| Item 5 (trouble starting things) | 200-006, age 29: *“This one is asking whether, uh, you've had any difficulty beginning new projects, so whether it's doing something around the house or going and doing something outside, uh, just kind of getting the energy to get up and do it.”*  200-007, age 36: *“I took that as like my motivation to want to get up and start something, for instance, the laundry; just looking at it, would I want to get up and start it.”* |
| Item 6 (trouble finishing things) | 600-004, age 66: *“You may be in the middle of, um, maybe a project, or I would hope not like dinner, but you just don’t feel like finishing it. You have no desire to finish it. You just are done.”*  700-001, age 44: “*If I do start something, do I have trouble bringing it to fruition?”* |
| Item 7 (have energy) | 200-001, age 61: *“Energy. It's having the oomph to go out and do things.”*  300-007, age 50: *“I have the energy. I have the, um, ability, I have the, um—that's just like motivation, um, initiative, um, as my mother would say, the gumption.”* |
| Item 8 (usual activities) | 100-004, age 19: *“So that one's just asking like how—how does your fatigue, like, how does it affect, you know, your daily—your routine.”*  200-006, age 29: *“Whether or not your fatigue is preventing you from doing anything that you would typically do throughout the week.”* |
| Item 9 (sleep during the day) | 600-008, age 31: *“I need to sleep during the day is what that one says, and I do frequently find that I become so fatigued throughout the day that I can’t function any longer, and I do end up having to sleep, because if I don't sleep, then I’ll just end up what I refer to as like being deliriously tired, where you can’t think straight and you literally can’t even sit and read a paragraph because your brain just isn’t having it.”*  200-002, age 33: *“I need sleep or rest, a nap, during the day.”* |
| Item 10 (too tired to eat) | 100-004, age 19: *“That's basically just how—how does your fatigue affect your eating habits?”*  200-006, age 29: *“It's asking whether or not you have any difficulty eating due to fatigue.”* |
| Item 11 (need help) | 700-006, age 34: *“If I need help doing activities around the house. Sometimes I do.”*  600-007, age 20: *“Like you need assistance, I don’t know, maybe somebody brushing your hair or, um, like you need somebody else to, to cook or to set out clothes for you or something like that.”* |
| Item 12 (frustration from being too tired) | 200-012, age 56: *“It’s asking me how frustrated I am where I can’t do the things that I want to do or need to do, and I have to ask for help or whatever, and that’s frustrating that you can’t do it yourself.”*  700-008, age 56: *“Being frustrated about being tired and not being able to do the things you want.”* |
| Item 13 (limited social activity) | 100-004, age 19: *“That one is how your social activity is affected by your fatigue, and it's easy to understand and easy to answer.”*  700-007, age 46: *“Basically, are you not doing things you normally do with your friends and family? Even if it's just going out to dinner somewhere, or, you know, meeting friends for coffee or whatever, you just can't bring yourself to do it.”* |

Abbreviations: FACIT-F, Functional Assessment of Chronic Illness Therapy–Fatigue

**Supplemental Table 7. Cognitive Debriefing Results for Overall CD Symptom PGRS, Overall CD Symptom PGIC, PGIS-Fatigue, and PGIC-Fatigue**

| **Items** | **Understanding/interpretation summary** |
| --- | --- |
| **Overall CD symptom PGRS** | All participants understood the overall CD symptom PGRS as intended. No concerning issues were reported. Twenty-seven participants (77%) indicated that bowel urgency was a factor in their response to the PGRS; 12 mentioned this spontaneously, and 15 confirmed when probed. Other symptoms that patients considered (all mentioned spontaneously) included abdominal pain (n = 18; 51%), stool consistency (n= 11; 31%), number/frequency of BMs they had had (n = 9; 26%), and fatigue (n = 6; 17%). |
| **Overall CD symptom PGIC** | All participants understood the overall CD symptom PGIC as intended, and no concerning issues were reported. Seven participants (20%) reported thinking about abdominal pain or cramping, four (11%) thought about the number/frequency of BMs, and four (11%) reported thinking about bowel urgency. Ten participants (29%) spoke more generally about considering different medications they had been on and comparing how they felt at various points to how they felt at the present time. |
| **PGIS-Fatigue** | Thirty-four participants (97%) indicated that the PGIS-Fatigue item was clear in meaning. The remaining participant had difficulty with most of the questionnaires, indicating that they had trouble reading “big words.” When asked about the timeframe they were asked to consider, 29 participants (83%) noted that the seven-day recall was clear and appropriate. Three participants (9%) suggested that a shorter (24-hour) recall period would be more appropriate. |
| **PGIC-Fatigue** | Overall, all participants indicated that the PGIC-Fatigue was clear in meaning and easy to answer. Three participants (9%) indicated that the question was somewhat confusing, but this was because they were not taking any “study medications.” Three participants (9%) suggested rewording the item slightly. |

Abbreviations: BMs, bowel movements; CD, Crohn’s disease; PGIS, Patient Global Impression of Severity; PGIC, Patient Global Impression of Change; PGRS, Patient Global Rating of Severity

**Supplemental Figure 1.** **Most Relevant FACIT-F Items: Part A-Qualitative Interviews**

Abbreviations: FACIT-F, Functional Assessment of Chronic Illness Therapy–Fatigue

**Supplemental Figure 2. Least Relevant FACIT-F Items: Part A-Qualitative Interviews**

Abbreviations: FACIT-F, Functional Assessment of Chronic Illness Therapy–Fatigue

**Supplemental Figure 3.** **Most Important FACIT-F Items for Improvement: Part A-Qualitative Interviews**

Abbreviations: FACIT-F, Functional Assessment of Chronic Illness Therapy–Fatigue
